# Analysis of noise and bias errors in intelligence information systems

**DOI:** 10.1002/asi.24707

**Published:** 2022-08-19

**Authors:** Ashraf Labib, Salem Chakhar, Lorraine Hope, John Shimell, Mark Malinowski

**Affiliations:** ^1^ Portsmouth Business School University of Portsmouth Portsmouth UK; ^2^ Centre for Operational Research & Logistics University of Portsmouth Portsmouth UK; ^3^ Department of Psychology University of Portsmouth Portsmouth UK; ^4^ Polaris Consulting Limited TP Group plc Farnborough UK

## Abstract

An intelligence information system (IIS) is a particular kind of information systems (IS) devoted to the analysis of intelligence relevant to national security. Professional and military intelligence analysts play a key role in this, but their judgments can be inconsistent, mainly due to noise and bias. The team‐oriented aspects of the intelligence analysis process complicates the situation further. To enable analysts to achieve better judgments, the authors designed, implemented, and validated an innovative IIS for analyzing UK Military Signals Intelligence (SIGINT) data. The developed tool, the Team Information Decision Engine (TIDE), relies on an innovative preference learning method along with an aggregation procedure that permits combining scores by individual analysts into aggregated scores. This paper reports on a series of validation trials in which the performance of individual and team‐oriented analysts was accessed with respect to their effectiveness and efficiency. Results show that the use of the developed tool enhanced the effectiveness and efficiency of intelligence analysis process at both individual and team levels.

## 1 INTRODUCTION

### 1.1 Noise and bias errors in intelligence information systems

An intelligence information system (IIS) is a special kind of information systems (IS) devoted to satisfying the unique intelligence functions and whose main users are intelligence analysts with a certain department, section, service, or agency (Achkoski & Trajkovik, 2011). A successful IIS should fully support all the activities within the intelligence cycle through which information is collected and assembled, raw information is transformed into processed information, analyzed, and made available to users. The basic and traditional intelligence cycle is composed of five main phases: planning and direction, collection, processing, analysis, and dissemination. We consider the analysis phase to be a complex intellectual process in which professional and military intelligence analysts play a key role, as they are able to put together diverse pieces of information and place them in a context which is useful for decision‐makers. Thus, in addition to organizational factors (e.g., structure and philosophy, team resources and administrative support, and communication strategies) that play a crucial role in determining the success of IS implementation (Yeoh & Popovič, 2016), having well‐trained professional intelligence analysts should also be prioritized by IIS stakeholders. Analysts face a number of challenges and they must tailor their work to be timely and relevant to decision‐makers, often in an environment in which the consequences of inaccuracy can be grave (Hare & Coghill, 2016). These challenges are the result of a combination of several characteristics related to individuals, organizations, and society, as well as the intelligence analysis process itself. These challenges are particularly important in the military intelligence context, which is the main focus of this paper.

Information overflow and diversity is a major challenge to military intelligence analysis, as analysts are typically required to process large volumes of a variety of data types in a timely manner so as to detect potential security threats (see, e.g., Duvenage, 2010; Hutchins et al., 2007; Treverton & Gabbard, 2008). The effect of information overflow and diversity on intelligence was recognized as early as the year 2000, when most of the current social media were nonexistent and when Berkowitz & Goodman (2000, p. 2) wrote that the “information revolution may be the single most important factor affecting intelligence today.” Successfully detecting potential security threats relies on consistent judgments (i.e., identical cases should be treated similarly, if not identically) by intelligence analysts in order to efficiently process the data and effectively identify useful information. In most instances, the analyst will be trained to recognize typical threat characteristics when reviewing data. For example, the analyst will review data to identify individual or group level suspicious activity or communications, which may be indicative of larger‐scale criminal or terrorist activities. It is vital that analysts, often operating as part of a team, be able to make consistent judgments about the value of this information, so it can be quickly extracted, enabling the appropriate security and counter‐terrorism measures to be taken.

Unfortunately, research and historical evidence (DeRosa, 2004; Fischhoff & Chauvin, 2011; Heuer Jr. & Pherson, 2014; Reyna et al., 2014) have shown that analysts' judgments are often inconsistent, due to a number of factors, including the sheer mass of data (DeRosa, 2004; Reyna et al., 2014), the variation in the types and nature of the intelligence information (Fischhoff & Chauvin, 2011), the lack of evidence presented (DeRosa, 2004), and the time pressures the analyst is operating under (Heuer Jr. & Pherson, 2014). Human cognitive limitations make up another important factor that can explain why analysts are likely to make inefficient and inconsistent decisions (Kim et al., 2020). Consequently, analysts will often take decisions that deviate significantly from those of their peers, from their own prior decisions, and from training rules that they themselves claim to follow (Kahneman et al., 2016). Such inconsistency is mainly due to two types of errors: noise and bias. Here, “noise” is defined as the variability of judgments or inconsistent decisions, whereas “bias” is defined as consistent diversion from the target (Adame, 2016; Hammond et al., 2006; Kahneman et al., 2016, 2021; Satopää et al., 2021). These errors complicate the process of intelligence analysis and can result in key pieces of data being misclassified or overlooked with potential implications for the recognition of security threats (Heuer Jr., 1999; Reyna et al., 2014).

In studying the effect of noise and bias errors on the accuracy of a decision, Kahneman et al. (2016) distinguished four possible outcomes: (a) an accurate decision (i.e., no noise, no bias); (b) noise, when there is variability within the judgments of different decision makers (or from the same decision maker on the same data); (c) bias, when judgments are similar but not correct (consistently wrong); and (d) noise and bias, when both (b) and (c) occur. Within the military intelligence analysis context, noise errors occur when intelligence reports are judged differently by different intelligence analysts. In the same context, bias errors occur when intelligence reports are wrongly judged by the different intelligence analysts, but they are consistently wrong: in other words, there is a consensus on the wrong interpretation or decision based on the available data. Noise and bias errors can occur simultaneously, leading to additional potential implications for the recognition of security threats.

The situation is further complicated when considering team‐based decision making, as opinions and unintentional (or intentional) biases within a group can lead to inconsistency and a lack of consensus. In this respect, Montibeller and von Winterfeldt (2015, 2018) consider that some cognitive biases may be exacerbated at the group level (or alleviated in some cases). Jones and Roelofsma (2000) suggest that group biases (namely false consensus, groupthink, group polarization, and group escalation of commitment) have a strong potential to affect team decisions and tend to be associated with those decisions that are important or novel and are exacerbated by time pressure and high levels of uncertainty. Within intelligence contexts, communicating pertinent information (e.g., relevant reports with crucial information about a planned attack) identified by one member of the analyst team to other team members is often difficult due to the speed and volume of incoming reports. Thus, and as recognized by several authors (e.g., Hastie, 2011; Kerr et al., 1996; Kerr & Tindale, 2011), team‐based decision making, or the analysis of input from several intelligence analysts, presents a challenge to modern intelligence analysis due to the inherent inconsistency that individual as well multiple opinions and inferences can lead to.

### 1.2 Information behavior and digital tools to inform the design of IIS and decision making

In addition to supporting the identification and correction/reduction of inconsistency related to noise and bias errors, an effective IIS should also take into consideration the research on information behavior for a more effective design of IIS and enhanced decision making. Information behavior aims to understand human behavior related to the search, retrieval, and use of information. It relies on different research methodologies grounded in different research paradigms, including psychology, sociology, and education (Waller et al., 2015). Information behavior can also enhance the design and implementation of decision tools, as shown by several studies (see, e.g., Allen et al., 2019; Huvila et al., 2016, 2022). Although information behavior research is well established from a theoretical point of view, there is a real gap with respect to its practical implication, mainly on the design of technological solutions for searching, retrieving and using information. In this respect, Huvila et al. (2021), through three example contexts, investigated the gaps between information behavior and practices and information systems design. They concluded that information behavior should go beyond the specific contexts and assumptions of the designer of an information system, to support the variety of ways information can be sought and used. This gap is also confirmed by Mishra et al. (2015), who concluded that information is often used to justify a decision rather than to support the process of making it. Our approach is intending to contribute to this gap as it links the behavior of security analysts with respect to noise and bias and attempts to measure these concepts through a developed IIS. Additionally, Huvila et al. (2021) identify safety‐critical environments as an area worth of investigating how information behavior and practice (IBP) and information system development (ISD) can interact. We argue that both safety and security are similar in that they both deal with risk and hazards, and that the main difference between both fields is in the intent (Schmidt et al., 2021).

Another important aspect to consider within the military intelligence context is the role of digital tools in the analysis of intelligence data and how they can support and improve decision making in this context. The authors advocate that digital tools can surely contribute to decision making. There is a large body of literature (see, e.g., Allen, 2011; van der Vegt et al., 2021) on decision making and the use of digital tools. For example, experimental research conducted by van der Vegt et al. (2021) in the medical domain indicates that effective “interpretation” ability is more important than having access to an improved search engine capabilities. Here interpretation is defined as from within documents and across them. Our tool attempts to contribute to such interpretation capabilities, in a military security domain. Furthermore, Chang et al. (2018) and Kahneman et al. (2016) assert that algorithms and digital tools are more effective than human beings in repetitive decision making. Although Kahneman et al. (2016) support the idea that algorithms often lead to a reduction of noise and bias, they also view the application of algorithms as a radical solution since they are sometimes politically or operationally not feasible. Furthermore, a growing research suggest that AI and algorithms should be used in caution as this may lead to algorithmic/automation bias (e.g., Ferguson, 2017; Lyell & Coiera, 2016; Parasuraman & Manzey, 2010; Rai et al., 2019; Sen et al., 2020). For example, Rai et al. (2019) assert that digital platforms are likely to align well with AI agents (such as speed, accuracy, etc.), but human competencies correspond better to other tasks.

The present authors support the idea that understanding information behavior and the use of digital tools and algorithms can enhance decision making in the intelligence context, but we also advocate that the design of these tools should strongly rely on the guidance of experienced intelligence analysts.

### 1.3 Design, implementation, and validation of an IIS to support the analysis of signals intelligence reports

Within this military context, the present authors have designed and implemented an innovative IIS that supports the five phases of the intelligence cycle. The developed tool, the Team Information Decision Engine (TIDE), was specifically designed to focus on the analysis of Signals Intelligence (SIGINT). SIGINT involves collecting intelligence information from electronic signals, communications and information systems of a given target and providing it to policing and military intelligence professionals. SIGINT reports contain a number of basic attributes (namely time, date, frequency, transmitter, and receiver). Each intelligence report also includes a Gist statement, prepared by a first line analyst. The acronym “Gist” (from Generating Interactions between Schemata and Texts), coined by Cunningham (1982), refers to a summarizing strategy used to improve student learning. In the considered intelligence context, a Gist statement corresponds to a summary, describing the “core” of what was discussed between the target subjects. Intelligence reports may also contain operator comments, that is, initial interpretations by first line analysts, such as the identification of code names and cover terms.

#### 1.3.1 Standard process for assessing SIGINT reports

The assessment of SIGINT reports involves three lines:

1. First line analysts, who are analysts on the ground. Their main task is the collection of intelligence information through different interception and communication tools. The information collected by the first line analysts is then reported in intelligence reports with specific structures and forwarded to headquarters.
2. Second line analysts are senior analysts located at headquarters. Their main task is to analyze the intelligence reports forwarded by first line analysts, typically allocating a risk rating to each report (e.g., a rating ranging from 1 [*no risk*] to 5 [*very high risk*]). At the end of their mission, second line analysts send Intelligence Summary (INTSUM) reports to their central command.
3. Third line analysts are central command agents receiving the summary reports, based on which they identify appropriate actions or mandate further investigations.

A report could reveal an actual threat (e.g., a terrorist plot) or not; it is up to the second line analysts to decide, individually, which reports should be dismissed and which ones should be investigated further. To this end, each second line analyst in the team scores each of the intelligence reports on a Likert scale from 1 to 5, representing the risk, as estimated by the analyst, associated to that report. The scores need to be somehow aggregated to come to the final decision on which reports should be pursued and which ones should be dismissed.

#### 1.3.2 Definition of noise and bias errors in the SIGINT context

**FIGURE 1:**
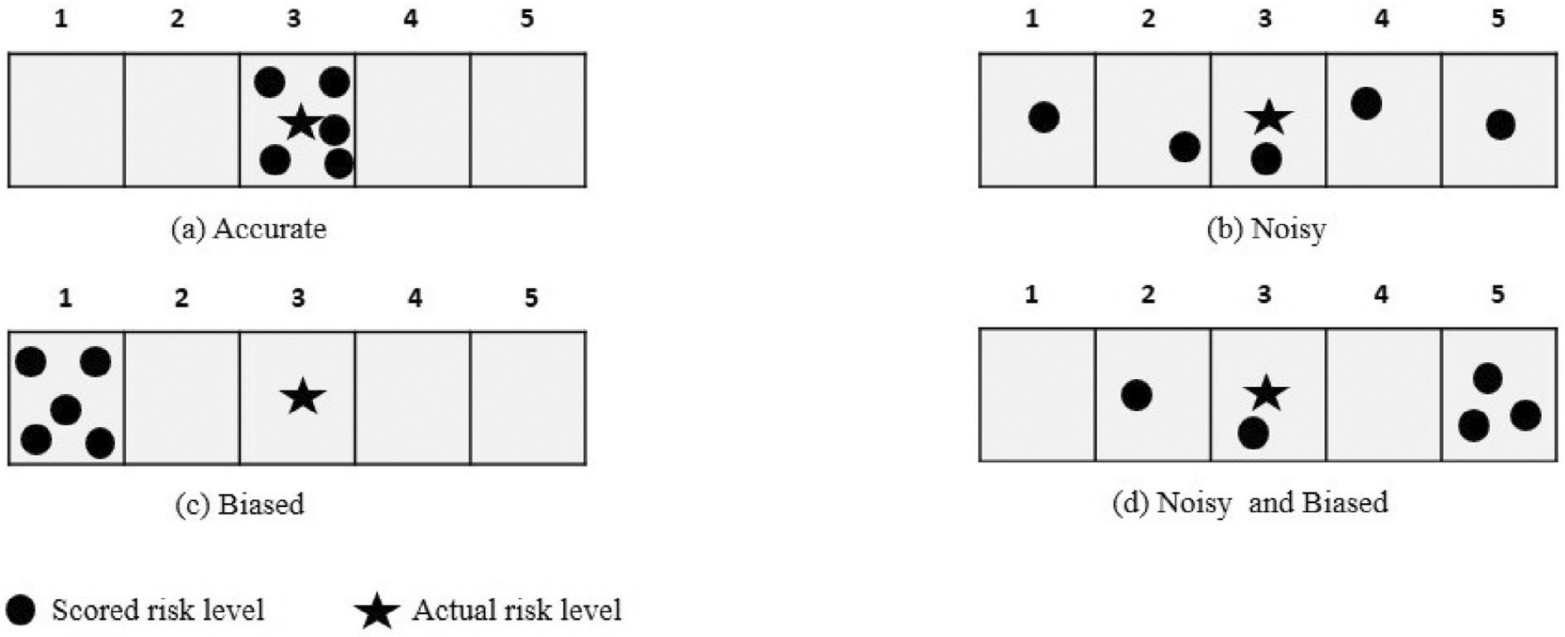
Noise and bias situations

Building on Kahneman et al.'s (2016) work, the present authors have formulated four possible outcomes in the considered military intelligence context, as illustrated graphically in Figure 1. In this figure, a Likert risk scale of five levels has been assumed, where level 5 is the highest risk level. Decisions (indicated by • in Figure 1) in situation (a) are accurate as the scores provided by different analysts are accurate, since they are close to the actual score. The other three situations are inaccurate but in distinct ways. In situation (b), the decisions are noisy, since the scores are widely scattered around the actual score (indicated by ⋆ in Figure 1). In situation (c), the decisions are biased, since they miss the actual decision but are clustered together. In situation (d), the decisions are both noisy and biased. Note here, as in Kahneman et al. (2016), that no hypothesis is made that the quality of judgment is measurable. Additionally, it is only by chance that situation (b), which is just noisy, or (d), which is a combination of noise and bias, could lead to situation (a), where we hit the right target by pure luck. Also in the case of situation (b) there is no need to know where the actual risk level score (indicated by ⋆) is located.

Within the considered military intelligence analysis context, noise errors occur when the intelligence reports are judged differently by different intelligence agents. The level of noise will increase with the distance between the risk levels of the target reports as specified by the different agents. In the same context, bias errors occur when the intelligence reports are wrongly judged by the different intelligence agents, but they are consistently wrong; in other words, there is a consensus on the wrong interpretation or decision based on the available data. Noise and bias errors can occur jointly, potentially leading to additional problems for the identification of security threats.

A noise error can be measured through dispersion statistics or in terms of the distances between the scores. A bias error can be measured in terms of the distance from the actual score. Here, we generally need to know the actual answer in order to measure bias (Kahneman et al., 2016). However, Satopää et al. (2021) argue we can rely on historical decisions to estimate and correct bias errors. The automatic correction of bias errors is difficult and may complicate the situation (adding more bias or noise). Generally, a possible solution to reduce the effect of bias errors is training in order to recalibrate judgments. In this respect, Biais and Weber (2009) find that providing the appropriate learning to hindsight‐biased analysts will help them to reduce volatility and improve performance.

#### 1.3.3 TIDE validation

TIDE was successfully demonstrated within an experiment that used volunteers, acting as intelligence analysts, and SIGINT data. The objective of this paper is to report the experimental validation phase for TIDE. For the purpose of evaluation, a series of measures have been used for evaluating the reduction in time spent on scoring, the quality of the scoring, and noise and bias errors at the individual and team levels. These proposed measures are straightforward, easily reproducible, and, most important, well accepted by those involved.

### 1.4 Organization of the paper

The rest of this paper is organized as follows. Section 2 provides a brief overview of TIDE. Section 3 details experimental design and evaluation measures used to assess TIDE. Section 4 analyses and discusses the results. Section 5 concludes the paper.

## 2 TEAM INTELLIGENCE INFORMATION SYSTEM

The TIDE tool has been developed for a UK Military SIGINT context to enable real‐time analysis of dynamic intelligence information, which will increase the rate at which situational awareness is developed. TIDE was designed to help second line analysts to process large volumes of data by capturing the rules and attributes by which they prioritized the data. This information was then used to dynamically help the analysts filter and prioritize intelligence reports, enabling them to extract more intelligence information more efficiently. The decision tool supports a team of analysts working on a common mission. Each can focus on different data sets to assess and score. TIDE works in the background to monitor their individual analyses and blend them into a team prediction based on all available data, so the whole team benefits from each other's analyses automatically. Accordingly, the tool enables a more rapid generation of situational awareness by blending the analyses of multiple analysts working simultaneously into a single view. In this way, pertinent information found by analyst A will feed into the situational awareness of analyst B automatically without the need for direct communication.

### 2.1 Architecture of TIDE

**FIGURE 2:**
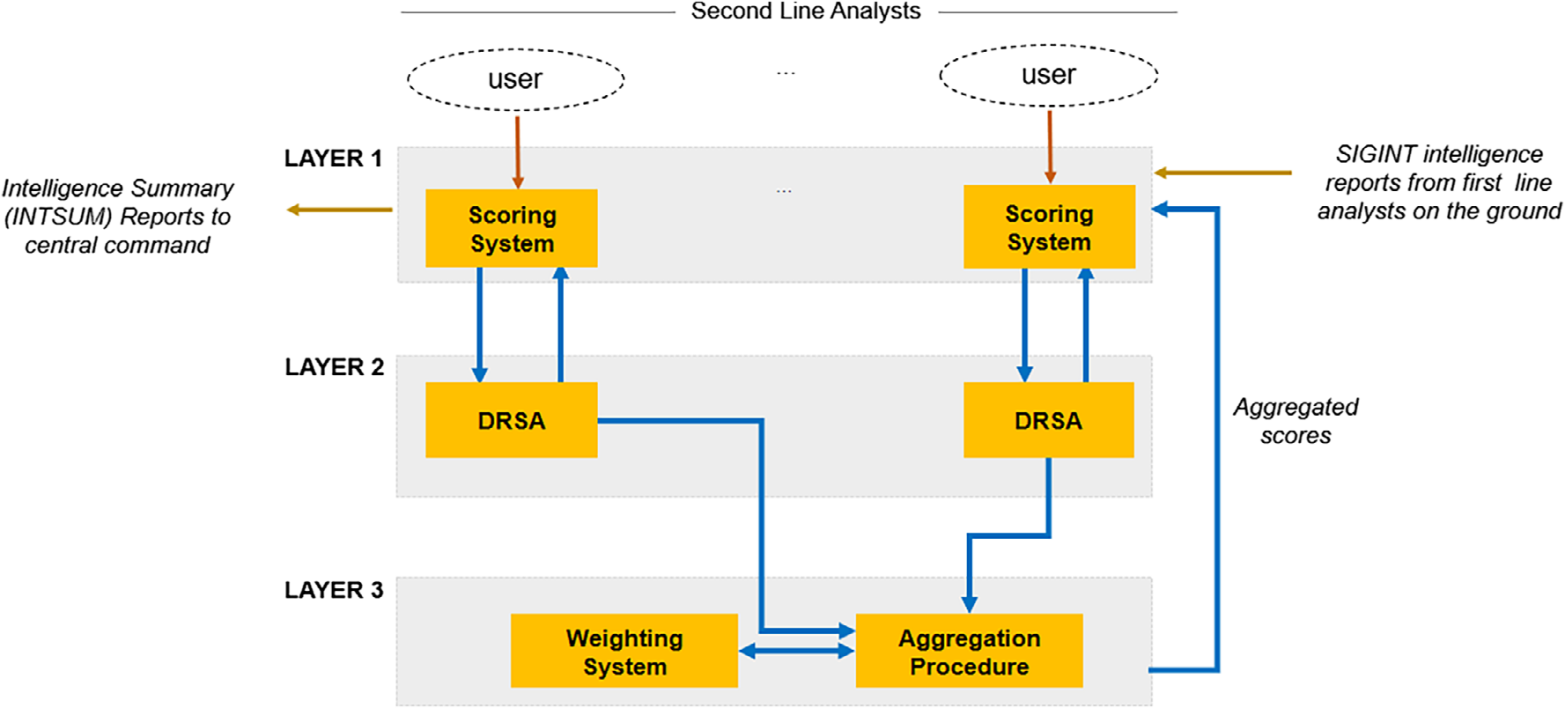
TIDE architecture

The conceptual architecture of TIDE is shown in Figure 2. TIDE follows a Client–Server Model architecture, where the clients are the second line individual analysts who can access the resources and services provided by the central server, accessible by central command. The decision tool is structured into three layers: (a) a scoring and reporting layer, (b) an individual assessment layer, and (c) a team aggregation layer (see Figure 2). A brief description of these layers follows.

The first layer constitutes the main interface of TIDE. Each second line analyst has access to her/his own private scoring and reporting main interface, enabling her/him to individually score intelligence reports. At the end of their mission, second line analysts should report their findings in an intelligence summary report (referred to as INTSUM) and send it to central command. The scores of the intelligence reports are dynamically updated to monitor and capture the judgments of the analysts and the team. Hence, the second line analysts have access to two types of judgments: (a) the scores obtained by applying the preference learning method (embedded in the second layer) using their own input data, and (b) team scores obtained by using an aggregation procedure (embedded in the third layer).

The second layer relies on a preference learning method, namely the Dominance‐based Rough Set Approach (DRSA) (Greco et al., 2001; Słowiński et al., 2009; Słowiński et al., 2002), to capture and reflect individual analyst judgments and behavior. The working principle of DRSA is similar to those of classical machine learning methods: it uses a subset of the data (here, the intelligence reports) to generate decision rules permitting the generalization of the preference information (which here takes the form of the scores assigned to the reports) to the whole data set (all intelligence reports). The use of DRSA permits TIDE to deduce relevant analyst insights from a subset of the intelligence reports, which can in turn be applied to predict the relevance of unseen intelligence reports. To compute the individual scores of the intelligence reports, the DRSA uses basic attributes of the intelligence reports (e.g., time, date, frequency, transmitter, receiver, etc.) and a set of additional attributes extracted in accordance with the search terms specified by the analyst. Three types of search terms can be specified by the analysts: names, places, and keywords. These categories of search terms are used to derive three score attributes for names, places and keywords, to enable a more accurate classification of the intelligence report without impacting significantly the processing time. The score calculation is further enhanced by using a weighting factor to allow analysts to prioritize keywords, with those words occurring at the top of the lists carrying more weight than those at the bottom.

The third layer draws on an innovative aggregation procedure (Chakhar et al., 2016) that allows the tool to incorporate group decision making to better reflect a team of intelligence analysts. The team judgment is obtained by combining multiple analyst judgments using the majority principle and veto effect. The majority rule is based on decisions taken by a majority vote and the veto effect is based on decisions taken by a minority vote. The majority with veto rule is implemented through the two following measures: (a) a concordance index representing the combined “weight” of the analysts who agree on the assignment of intelligence reports; and (b) a discordance index representing the combined “weight” of the analysts who do not agree on the assignment of intelligence reports. The computing of these measures uses a comprehensive weighting system that reflects the expertise and the quality of the individual assignments given by each analyst. Indeed, the aggregation procedure has the ability to give different team members different “weights,” which means their opinions hold more sway in the collective score.

TIDE has been designed in such a way that team judgments are automatically computed in a timely fashion and shared with all analysts. Thus, the aggregated scores of intelligence reports will play the role of group feedback. We believe that the timely availability of group feedback will help in reducing inconsistency (especially noise error) at the team level. This is mainly due to the use of majority with veto rule.

TIDE is innovative with respect to three main contributions:

1. Preferences and behavior of analysts are explicitly included within the scoring process (through the use of a preference learning method). Classical machine learning methods cannot properly take into consideration the preferences of analysts (since attributes and classes are assumed to be without preference order).
2. Individual scores are objectively combined through the use of majority/veto effect rule along with the analyst's power. Most of the existing approaches either use a weighted‐like method or simple aggregation rules (such as minimum, maximum, or average values) to compute the aggregated scores.
3. Objectively taking into account the levels of expertise of the analysts through the quality of their scoring. Most of the existing methods either do not use any weighting system, or use predefined power levels.

In addition, the measures introduced in the next section and used for evaluating TIDE (with respect to reducting the time spent on scoring, the quality of the scoring, and noise and bias errors at the individual and team levels) are straightforward, easily reproducible, and, most important, well accepted by those involved.

### 2.2 Specification of search keywords

The intelligence reports contain a number of basic attributes (e.g., time, date, frequency, transmitter, receiver, etc.). These basic attributes are applied to highlight the initial reports of interest by exploiting a dominance relation, which is the most elementary preference information. The dominance principle, which can be seen as monotonicity constraints, is stated as follows for our military application: “If intelligence report *x* is at least as risky as intelligence report *y* with respect to all basic attributes, then intelligence report *x* should be classified as being at least as risky as intelligence report *y*.” The analyst can also specify different “terms of interest,” for example, keywords and geographical areas, based on the intelligence briefing and objectives. To generate additional attributes, each intelligence report would need preprocessing, in accordance with the search terms specified by the analyst. Various methods were explored for deriving additional attributes to reflect search terms like keywords. This varied from a simple count attribute for each user‐specified keyword to a single total keyword count attribute for all keywords. The former would be likely to enhance the process of classifying the report, and the accuracy of the predictions, but would potentially be time‐consuming to run, while the latter would be likely to lead to less accurate predictions but be more efficient in terms of processing time.

A compromise was reached, whereby three simple categories of keywords could be specified by the user (names, places, keywords) and preference ordered within a list. These categories would be used to derive three score attributes for names, places and keywords, to enable more accurate classification but not have a significant effect on processing time. The score calculation was further enhanced by using a weighting factor to allow analysts to prioritize keywords, with those words occurring at the top of the lists carrying more weight than those at the bottom.

### 2.3 Scoring of intelligence reports at individual level

Scores at the individual level are computed by the DRSA based on exemplary scores provided by the analysts and serving as a learning set for the DRSA. Accordingly, the analysts should review and prioritize a set of interesting reports and then assign a risk level to each of these reports. This is the learning set. The risk levels are expressed on an ordinal scale. In the considered military application, we used a Likert scale of five levels, from 1 to 5, where 5 is the highest risk level. The results of this process constitute a decision table. The entries of the decision table are attribute–value pairs. The values for these attributes are automatically extracted from the characteristics of the intelligence reports (such as location, frequency, etc.) or computed based on the scoring of the intelligence reports by the analysts.

It was recognized that DRSA can generate rules with multiple decision states, that is, an unscored report could potentially be of multiple interest levels. However, to minimize confusion for the analyst, the decision was taken to present a single DRSA‐derived interest level only: the highest predicted interest score. Although this could result in reports being assigned a higher interest level than they actually ought to have (a false positive), it was felt that in an intelligence context, this was better than selecting a lower predicted interest (a true negative) and potentially missing critical intelligence.

### 2.4 Scoring of intelligence reports at team level

The scores at the team level are automatically computed by the aggregation procedure. The basic idea for computing the aggregate score relies on the majority rule and veto effect (i.e., minority respect), which originated from social choice theory and are now well established in decision making (see, e.g., Bouyssou, 2009). The majority rule is based on decisions taken by a majority vote and the veto effect is based on decisions taken by a minority vote (see, e.g., Schermers & Blokker, 2018). In Chakhar et al. (2016), majority and veto rule are implemented through the two following measures:

1. $C\left(R_{i},{\mathrm{Cl}}_{j}\right)$: a concordance index representing the combined “weight” of analysts who agree on the assignment of intelligence report $R_{i}$ to risk level ${\mathrm{Cl}}_{j}$.
2. $D\left(R_{i},{\mathrm{Cl}}_{j}\right)$: a discordance index representing the combined “weight” of analysts who do not agree on the assignment of intelligence report $R_{i}$ to risk level ${\mathrm{Cl}}_{j}$.

The computing of these measures uses a comprehensive weighting system that reflects the expertise and the quality of the individual assignments given by each analyst, as explained in the next subsection. The concordance index $C\left(R_{i},{\mathrm{Cl}}_{j}\right)$ measures the power of the coalition of analysts that assign intelligence report $R_{i}$ to risk level ${\mathrm{Cl}}_{j}$. The concordance index values are in range [0,1]. It takes 1 when there is a full agreement between analysts to assign a risk level of ${\mathrm{Cl}}_{j}$ to intelligence report $R_{i}$, and 0 when there full is disagreement about this assignment. The discordance index $D\left(R_{i},{\mathrm{Cl}}_{j}\right)$ measures the power of the coalition of analysts that are against assigning a risk level of ${\mathrm{Cl}}_{j}$ to intelligence report $R_{i}$. It takes 1 when there is a full agreement between analysts for nonassigning a risk level of ${\mathrm{Cl}}_{j}$ to intelligence report $R_{i}$ and 0 when there is a full disagreement about this nonassignment. Then, assigning intelligence report $R_{i}$ the risk level ${\mathrm{Cl}}_{j}$ occurs if and only if:

(1) $$\sigma \left(R_{i},{\mathrm{Cl}}_{j}\right)=C\left(R_{i},{\mathrm{Cl}}_{j}\right)\times D\left(R_{i},{\mathrm{Cl}}_{j}\right)\geq \lambda ,$$

where $\lambda \in \left[0.5,1\right]$ is the credibility threshold representing the minimum value for the credibility index $\sigma \left(R_{i},{\mathrm{Cl}}_{j}\right)$ for assigning an intelligence report $R_{i}$ the risk level ${\mathrm{Cl}}_{j}$. The assignment rule above ensures that intelligence report $R_{i}$ is assigned risk level ${\mathrm{Cl}}_{j}$ if and only if: (a) a majority of analysts, in view of their “weights,” support this assignment; and (b) none of the analysts who do not support this assignment express too strong disagreement. The credibility threshold λ value is domain‐specific. In the considered case study, the value of the credibility threshold λ was set to 0.75 to ensure more than a two‐thirds majority vote.

### 2.5 Weighting system

The aggregation procedure introduced in the previous section uses a comprehensive weighting system permitting the objective measurement of the “weights” of analysts based on the quality of their inputs during the scoring processes by considering:

- The quality of classification that measures the number of correct scores provided by each analyst.
- The accuracy of approximation that measures these scores for each risk level.

The quality of classification provides a global view of scoring process quality while the accuracy of approximation offers a more local view of scoring process accuracy. This double weighting system permits to model better the fact that some analysts are better in identifying risky SIGNT reports while others do better with low risky reports. The weighting system is formally defined as follows:

(2) $$\pi_{k}\left({\mathrm{Cl}}_{t}^{⋄}\right)=\frac{\gamma_{k}\alpha_{j}\left({\mathrm{Cl}}_{t}^{⋄}\right)}{\sum_{r\in_{j}^{⋄}}\gamma_{r}\alpha_{j}\left({\mathrm{Cl}}_{t}^{⋄}\right)+\sum_{r\notin_{j}^{⋄}}\gamma_{r}}.$$

Here, H is the set of analysts, $H_{j}^{⋄}=\left\{j:j\in H\;\text{and}\;\alpha_{j}\left({\mathrm{Cl}}_{t}^{⋄}\right)>0\right\},\;\gamma_{k}$, is the quality of classification of analyst k and $\alpha_{j}\left({\mathrm{Cl}}_{t}^{⋄}\right)$ is the accuracy of approximation of class ${\mathrm{Cl}}_{t}^{⋄}$ by analyst k.

## 3 EXPERIMENTAL DESIGN AND EVALUATION MEASURES

### 3.1 Experimental design

#### 3.1.1 Aim and description

The aim of TIDE is to help the intelligence analyst to be (a) effective, that is, to reach the right answer—a measure of effectiveness based on identification of critical target event information, measured by accurately scoring, and producing a summary report; and (b) efficient, that is, within the minimum time—an efficiency measure by which the decision is made (fewer reports assessed to reach the same conclusion). A series of trials involving simulated intelligence data were conducted to test the effectiveness and efficiency of TIDE. A description of these trials is shown in Table 1. For the purpose of analysis, the participants were organized into three types of groups, as follows:

1. Non‐DRSA group (group #2): This is a control group where participants have no access to the DRSA. The participants of this group have the same main interface as the following two groups, but there is neither DRSA‐based scoring nor the aggregated scores.
2. DRSA without group feedback (groups #1 and #3): This group has access to DRSA‐based scoring only. The aggregated scores are hidden from the participants of this group.
3. DRSA with group feedback (groups #4, #5, or #6): This group has access to DRSA‐based scoring as well as the aggregated scores.

**TABLE 1:** Characteristics of conducted trials

Trial	Group number	DRSA	Group feedback	Number of participants
1	1	Yes	No	7
2	2	No	No	8
2	3	Yes	No	7
2	4	Yes	Yes	7
3	5	Yes	Yes	8
4	6	Yes	Yes	8

All groups were given background briefings and training on their respective tools and given the necessary time to practice using the decision tool, before starting the experiments.

#### 3.1.2 Participants

The participants (N=45) involved in the different trials were lay volunteers acting as intelligence analysts. In the rest of this paper, the participants will be referred to as “analysts.”

#### 3.1.3 Scenario design

A test set of SIGINT data was generated, drawing on the team's experiences creating scenario data for the Ministry of Defence (MoD) SIGINT training exercises, which was supported by ex UK SIGINT/intelligence analyst Subject Matter Experts (SMEs). The scenario was designed to reflect a far right terrorist attack being planned in a Western European city and contained terrorist planning, logistics and reconnaissance cells. UK Electronic Warfare (EW) interception assets had captured radio transmissions of RED (criminals/terrorist), GREEN (military police) and WHITE (civilians) organizations over a 5‐day period and fed in their initial reports to a second line intelligence analysis cell. In the simulation, the second line analysts are overwhelmed with these data and need to process these reports to extract intelligence pertaining to the plans of RED.

#### 3.1.4 Test data generation

A total number of 441 examples of SIGINT reports were generated by an Excel spreadsheet and exported to individual .txt files to be used within TIDE. Each report contained a number of attributes, such as time, date, frequency, transmitter, receiver, and so forth. In order to test the ability of TIDE to help the analysts quickly focus on the RED reports containing the intelligence, the majority of the reports generated were of GREEN and WHITE nature.

The test data was modified to reflect typical SIGINT report formats, which included removing some attributes (e.g., to/from, frequency) and including mis‐spellings of names and places. Although this makes it more challenging to manually review the reports and extract intelligence, it was hoped this would improve the ability of DRSA to deal with a data structure with missing attributes.

### 3.2 Measuring total scoring time

The assessment of intelligence reports relies on a preference learning method, namely DRSA. As the working principle of DRSA is similar to those of classical machine learning methods, it will naturally reduce the time needed, as analysts are not required to score all the intelligence reports. Let n be the total number of reports received over a period T of time (e.g., duration of a session) and let $T_{R}$ be the average processing time required to analyze an intelligence report by a given analyst. An analyst without DRSA will then require an average total processing time of $n\times T_{R}$ to score all the reports. By using DRSA, the analyst needs to score only a subset of k<n reports on average: the remaining n−k reports will be assessed automatically using the decision rules deduced from the scoring of the k reports. Then, the reduction of total scoring time can be measured as follows:

(3) $$\text{Total scoring time reduction}=n\times T_{R}-k\times T_{R}.$$

In practice, analysts are often under pressure and a non‐DRSA analyst may not be able to assess all the reports during time period T, with potential implications for national security. This occurs when the average total processing time exceeds the allowed processing time, that is, $n\times T_{R}>T$.

### 3.3 Measuring the quality of scoring

The quality of the scores provided by each second line analyst can be evaluated through the quality of the classification by DRSA. The quality of classification is defined as the ratio of all correctly classified intelligence reports to all intelligence reports:

(4) $$\text{Quality}=\frac{n-b}{n}.$$

Here, n is the total number of intelligence reports and b (with b≤n) is the number of intelligence reports incorrectly classified. The quality of classification ranges over the interval [0,1], where 1 means a perfect classification, that is, all intelligence reports have been correctly classified, while 0 means a perfect misclassification, that is, all intelligence reports have been wrongly classified.

### 3.4 Measuring the efficiency and effectiveness of analysts

The efficiency of an analyst is computed as the number of reports (k) explicitly scored by this analyst divided by the number of received reports (m):

(5) $$\text{Efficiency}=\frac{k}{m}.$$

The effectiveness of an analyst can be evaluated by examining patterns of “hits,” “misses,” and “false alarms.” This can be achieved by analyzing the final scores of intelligence reports with actual scores computed by the authors based on the scenario used during the validation phase. In this application, correctly identifying intelligence reports with actual scores of 4 or 5 is considered as a hit while assigning a score of 1, 2 or 3 to an intelligence report of actual score of 4 or 5 is considered as a miss. It is considered a false alarm when an intelligence report which ought to be scored 1, 2 or 3 is scored 4 or 5. All these situations are summarized as follows:

- True Positives (TP) (i.e., Hits): The cases in which an analyst assigned 4 or 5 while the scores should have been 4 or 5.
- True Negatives (TN) (i.e., Correct Rejection): The cases in which an analyst assigned 1, 2, or 3 while the actual scores should be have been 1, 2, or 3.
- False Positives (FP) (i.e., False Alarms or Type I Error): The cases in which an analyst assigned 4 or 5 while the actual scores should have been 1, 2, or 3.
- False Negative (FN) (i.e., Misses or Type II Error): The cases in which an analyst assigned 1, 2, or 3 while the actual scores should have been 4 or 5.

It is important to stress that this mapping of risk levels will not affect the actual risk levels of the intelligence reports: it is only used for purposes of experimental analysis to evaluate the effectiveness of the analysts.

The effectiveness of the analysts can then be measured as follows:

(6) $$\text{Effectiveness}=\frac{\mathrm{TP}+\mathrm{TN}}{\mathrm{TP}+\mathrm{TN}+\mathrm{FP}+\mathrm{FN}}.$$

### 3.5 Measuring noise and bias errors at the individual level

Noise and bias errors at the individual level arise mainly when intelligence analysts take decisions that deviate from their own prior decisions (Kahneman et al., 2016). Noise errors can be identified by analyzing the scores successively provided by an analyst at different points of time during the same work session, or for the same project but in different time periods. Only the first case is considered in this paper. To measure the noise error, we will employ the well‐known nonparametric statistic, Kendall's tau (Kendall, 1938; Kendall & Gibbons, 1990), defined as follows. Let $\left(S_{1,t},S_{1,t^{\prime }}\right),\left(S_{2,t},S_{2,t^{\prime }}\right),\ldots ,\left(S_{n,t},S_{n,t^{\prime }}\right)$ be the scores of intelligence reports $R_{1},R_{2},\ldots ,R_{n}$ assigned at times t and $t^{\prime }$. Then, a pair of scores $\left(S_{i,t},S_{i,t^{\prime }}\right)$ and $\left(S_{j,t},S_{j,t^{\prime }}\right)$ is said to be

- Concordant if (a) $S_{i,t}<S_{i,t^{\prime }}$ and $S_{j,t}<S_{j,t^{\prime }}$ or (b) $S_{i,t}>S_{i,t^{\prime }}$ and $S_{j,t}>S_{j,t^{\prime }}$.
- Discordant if (a) $S_{i,t}>S_{i,t^{\prime }}$ and $S_{j,t}<S_{j,t^{\prime }}$ or (b) $S_{i,t}<S_{i,t^{\prime }}$ and $S_{j,t}>S_{j,t^{\prime }}$.
- Neither concordant nor discordant otherwise, that is, if none of the previous situations hold.

Kendall's tau $\tau_{t,t^{\prime }}$ can be applied with or without ties (see, e.g., Agresti, 2010). When ties are allowed, Kendall's tau between the scores at times t and $t^{\prime }$ is defined as follows:

(7) $$\tau_{t,t^{\prime }}=\frac{\left(n_{c}-n_{d}\right)}{\sqrt{\left(n_{0}-n_{1}\right)\left(n_{0}-n_{2}\right)}}.$$

Here, $n_{0}=n\left(n-1\right)/2$, $n_{c}$ is the number of concordant pairs, $n_{d}$ is the number of discordant pairs, and $n_{1}=\sum_{k}n_{k}\left(n_{k}-1\right)/2$, $n_{2}=\sum_{h}n_{h}\left(n_{h}-1\right)/2$ with $n_{k}$ and $n_{h}$ are the number of tied values in the *k*th and *h*th groups of ties in the first and second series of scores, respectively. Note that there are other methods for measuring noise, such as the one proposed by Kahneman et al. (2016), where a noise index is computed as the difference divided by the average of the pair.

Kendall's tau lies in the range $\left[-1,1\right]$. If the agreement between the scores at two different points of time is perfect (i.e., no noise error), τ is 1. If the disagreement between the scores is perfect (i.e., the scores are the reverse of each other and then all scores are noisy), it is −1. If the scores at two different times were independent, then we would expect it to be approximately zero.

Bias errors can be identified by analyzing the scores provided by an analyst at a given point in time with the actual threat level of the intelligence report. If we denote by $S_{i}^{*}$ $\left(i=1,\ldots ,n\right)$ the actual threat level of intelligence report $R_{i}$, then the bias errors between it and the scores at time t can be measured using Kendall's tau for the pairs $\left(S_{1,t},S_{1}^{*}\right),\left(S_{2,t},S_{2}^{*}\right),\ldots ,\left(S_{n,t},{S_{n}}^{*}\right)$. The description of the values of Kendall's tau in the range $\left[-1,1\right]$ still applies here, but it should include the scores at a given point in time and the deserved scores.

### 3.6 Measuring noise and bias errors at team level

Noise and bias errors at the team level are evaluated using the nonparametric statistic Kendall's W (also known as Kendall's coefficient of concordance) (Kendall & Gibbons, 1990). Let $S_{i,k}$ be the score of report $R_{i}$ by analyst k where there are n reports and m analysts. Then, Kendall's W for noise errors is defined as follows:

(8) $$W=\frac{12S}{m^{2}\left(n^{3}-n\right)}.$$

Here, $S=\sum_{i}{\left(S_{i}-\hat{S}\right)}^{2}$ with $S_{i}=\sum_{k}S_{i,k}$ is the total score given to report $R_{i}$, and $\hat{S}=1/n\sum_{i}S_{i}$ is the mean value of these total scores.

If Kendall's W is 1, there are no noise or bias errors over time. If W is 0, then there is no overall trend to the scores, and the scores may be regarded as essentially random. Intermediate values of W indicate a greater or lesser degree of unanimity between the scores.

The Kendall's W for bias error is defined in the same way but it should also include the actual threat level of report $R_{i}$ (which will give m+1 scores for each report).

## 4 RESULTS AND DISCUSSION

First, we present in Table 2 some general statistics (computed based on the results obtained at the end of the scoring process) about (a) the number and percentage of received reports, (b) the number of explicitly scored reports, and (c) the number of attributes. Based on the data in Table 2, we can conclude that: (a) analysts using DRSA scored many fewer reports (between 17.91 and 22.45%) than those with no DRSA (about 65%); (b) none of the non‐DRSA analysts scored all the reports; (c) non‐DRSA analysts used slightly more attributes (about 98 attributes) than those using DRSA (between 59 and 84 attributes); (d) there was no significant difference with respect to the number of attributes between analysts using DRSA without group feedback and those using DRSA with group feedback.

**TABLE 2:** General statistics

Trial	Group	Received reports		Scored reports		Number of attributes
		Number	%	Number	%	
1	DRSA without group feedback	441	100	94	21.41	59
2	Non‐DRSA	441	100	287	65.00	98
2	DRSA without group feedback	441	100	89	20.12	84
2	DRSA with group feedback	441	100	99	22.45	77
3	DRSA with group feedback	441	100	79	17.91	82
4	DRSA with group feedback	441	100	91	20.63	90

The measures introduced earlier have been used to evaluate the total reduction in scoring time, the quality of the classification, the efficiency and effectiveness of the analysts, and the noise and bias errors. Excepting the first measure, all measures were evaluated at regular intervals during the trials. A summary of the results is given in Table 3.

**TABLE 3:** Summary of noise and bias errors at individual and team levels

Trial	Group	Scoring time reduction	Quality of scoring	Efficiency	Effectiveness	Individual level		Team level	
						Noise	Bias	Noise	Bias
1	DRSA without group feedback	59.92	0.80	0.33	0.85	0.723	0.656	0.805	0.688
2	Non‐DRSA	‐	0.70	0.65	0.72	0.546	0.437	0.722	0.795
2	DRSA without group feedback	57.68	0.80	0.31	0.83	0.683	0.701	0.843	0.700
2	DRSA with group feedback	61.52	0.75	0.34	0.92	0.879	0.820	0.858	0.735
3	DRSA with group feedback	58.64	0.92	0.28	0.88	0.915	0.867	0.899	0.923
4	DRSA with group feedback	57.04	0.87	0.32	0.92	0.893	0.812	0.807	0.784

### 4.1 Total scoring time

Equation (3) has been used to compute the reduction of total scoring time, defined as the difference between the average total scoring time theoretically required to score n=441 intelligence reports and the average total scoring time of the reports actually scored by the analysts. The results of the non‐DRSA group (group #2) have been used to estimate the average processing time $T_{R}$ (used in Equation (3)) of a single report. This led to an average processing time of 0.32 min per report and an average required total processing time of 141.12 min. The reduction of total processing time is then computed through Equation (3). The results (see Table 3) show an average processing time reduction of about 58.96 min.

The reduction of the total scoring time is a direct consequence of the machine learning aspect of the DRSA, since analysts with DRSA support scored a reduced set of intelligence reports, rather than the systemic scoring of all intelligence reports for non‐DRSA users. An important remark concerns the minimum number of reports that should be scored by the analysts in order to enable DRSA to work properly. This is in fact not just specific to DRSA but also relates to all machine learning methods.

### 4.2 Quality of scoring

Equation (4) has been used to compute the quality of classification for all analysts. We note that the quality of classification for non‐DRSA analysts has been computed by the authors using the history of scored reports as learning set. The results in Table 3 indicate that the quality of classification of DRSA analysts was slightly better than those without DRSA. The results also indicate that there was no significant difference with respect to quality of classification between analysts using DRSA without group feedback and those using DRSA with group feedback.

### 4.3 Efficiency of analysts

**FIGURE 3:**
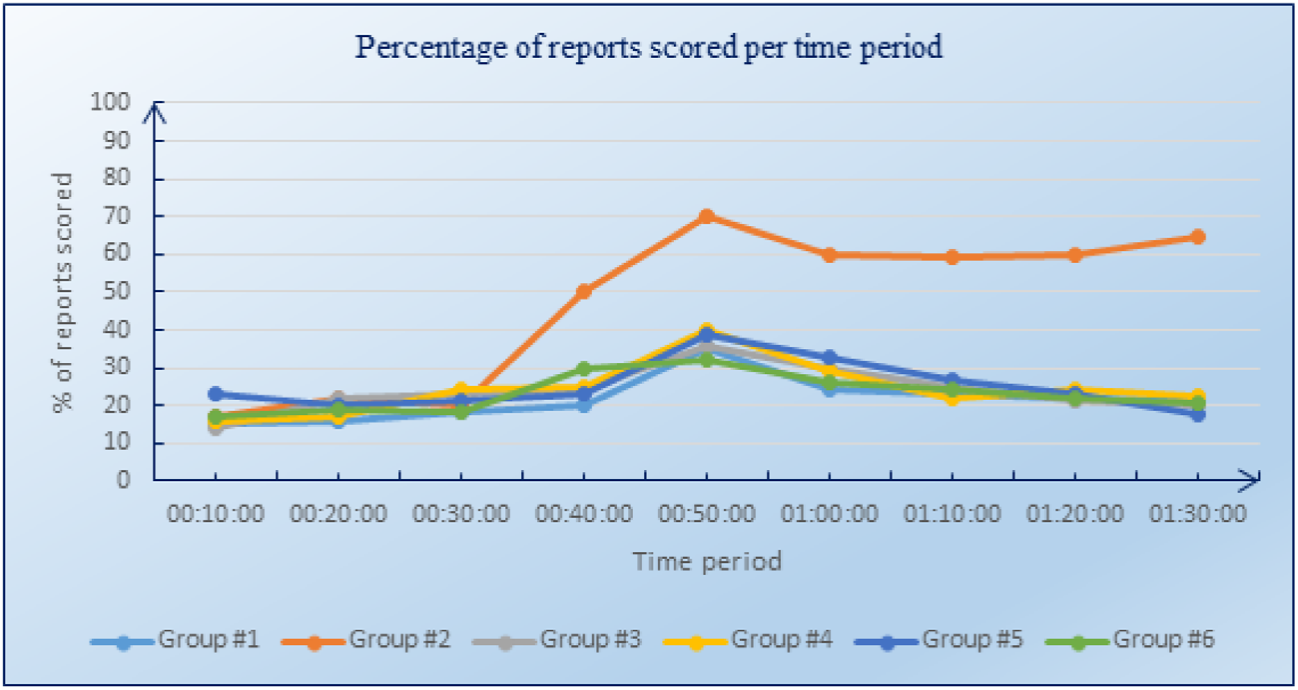
Evolution over time of the number of scored reports

The efficiency of analysts is computed in Equation (5) as the ratio of the number of reports scored (k) by an analyst during period time T to the maximum number of explicitly scored reports (m) during the same time period. The results (see Table 3) indicate an average number of 123 explicitly scored reports. We also evaluated the efficiency of analysts by analyzing the evolution over time of the number of reports scored. For this purpose, the data for the number of reports scored versus the available reports at each 10‐min time snapshot were collated. The chart in Figure 3 shows the average percentage figures at each time period for all groups of analysts. Note that the group numbers in this figure refer to the group numbers given in the second column of Table 1. The percentages were weighted equally for each analyst. As shown in Figure 3, overall, the non‐DRSA group read a much higher percentage of the available reports than did the DRSA groups. For the first 30 min, there is no clear difference between the non‐DRSA group and the DRSA groups. From 30:00 onwards, there is a distinct and consistent difference between the non‐DRSA group and the DRSA groups, with the non‐DRSA group reading around 50% of the available reports, and the DRSA group reading around 20% of reports (a reduction by 60%).

The analysis of the evolution over time of the number of reports read indicates that DRSA enabled the analysts to dismiss or filter out a lot of reports and to target the most salient reports. Results showed that analysts using DRSA, in comparison to analysts not using DRSA, scored fewer reports (18–22% vs. 65% respectively), and that none of the analysts scored all the reports. The difference between the DRSA and non‐DRSA groups seems to start after about 30:00. The most likely explanation for this is that it took time for the DRSA learning of the sets of keywords and ratings to mature to a state that it made a significant difference.

Filtering noninteresting intelligence reports will naturally improve the efficiency of the analysts (this is due to the reduced number of reports that have to be scored by the analysts). The rules inferred by DRSA from explicitly scored reports are automatically used to score all existing (or incoming reports).

### 4.4 Effectiveness of analysts

The effectiveness of analysts is evaluated by examining patterns of “hits” (i.e., True Positives), “misses” (i.e., False Negatives), and “false alarms” (i.e., False Positives) in the identification of important reports (i.e., reports scored either 4 or 5) by comparing the scores provided by the analysts with the actual threat levels. In this application, the actual threat levels have been defined based on information provided by the senior intelligence expert who designed the scenario. The effectiveness of analysts is measured through the scoring accuracy (see Equation (6)) and computed as the ratio of the number of True Positives plus the number of True Negatives to the total number of scored reports. As shown in Table 3, the effectiveness of the analysts for the different sessions at the end of the scoring exercise indicates that DRSA users are more effective than non‐DRSA users. Table 3 also shows that group feedback slightly improves the effectiveness of analysts.

**FIGURE 4:**
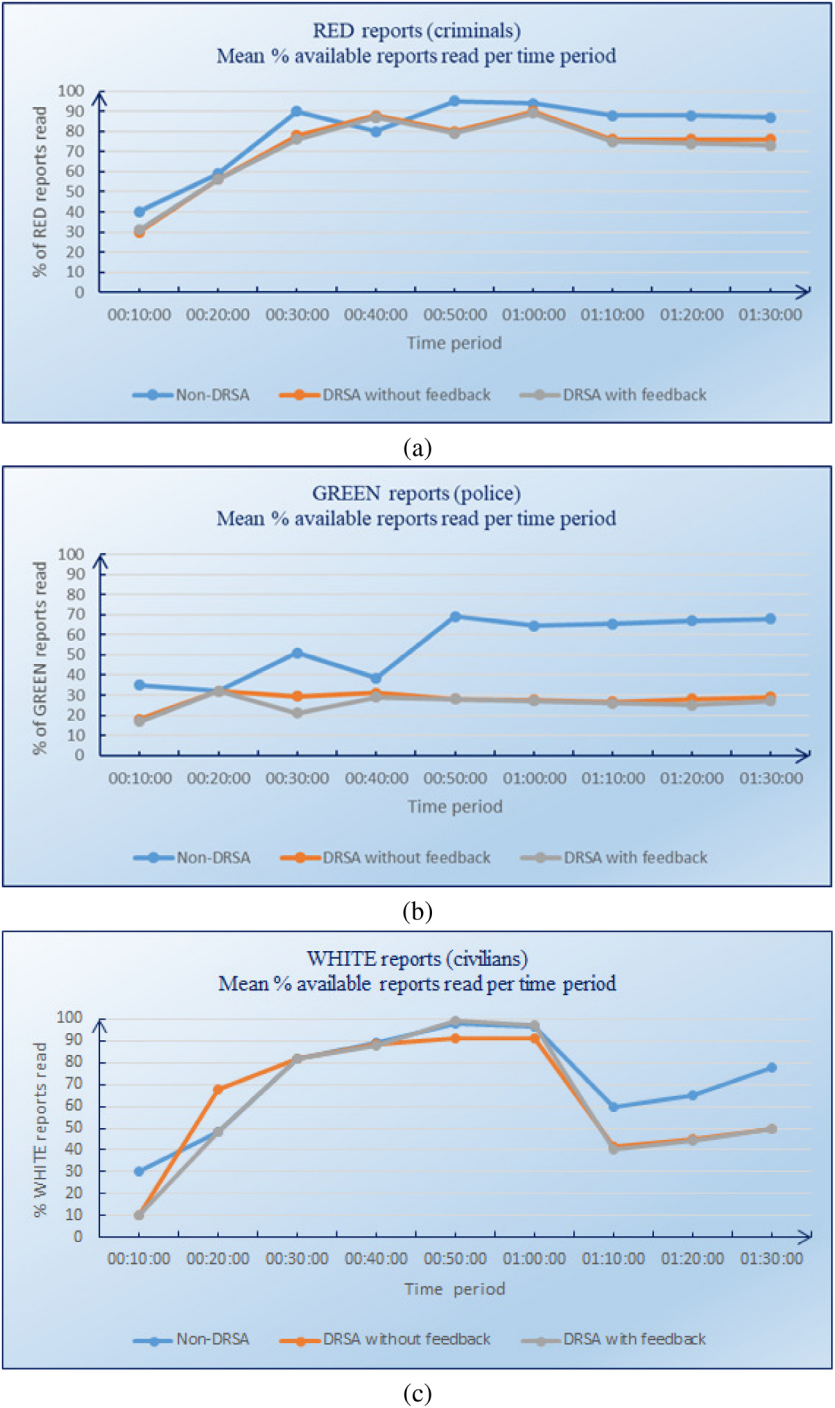
Evolution over time of RED (a), GREEN (b), and WHITE (c) reports read

We also evaluated the evolution over time of RED, GREEN, and WHITE reports read. The results are shown in Figure 4a–c, respectively. These figures indicate that the DRSA with feedback group performed slightly better than the DRSA without feedback group, and much better than the non‐DRSA group.

The improvement of the effectiveness of analysts is due to the automatic application of decision rules, which enables the elimination/reduction of the number of “misses” (i.e., False Negatives) and “false alarms” (i.e., False Positives). The improvement of effectiveness is also due to the “algorithmic” behavior of decision rules, which are applied with no need for human intervention. Indeed, several authors (e.g., Chang et al., 2018; Kahneman et al., 2016) have asserted that algorithms are more effective since they lead to the same decision if they are applied on the same or similar data while decisions specified by a human being may vary over time (e.g., as a result of time pressure, tiredness, loss of concentration, presence of external disturbances, work pressure). At this level, it is important to reiterate that AI and algorithms should be used in caution to avoid algorithmic/automation biases. The use of decision rules as in TIDE is much more flexible than the use of classical formal algorithms, since decision rules are inferred from the input of the analysts and also because the set of decision rules evolve over time, along with the cognitive behavior of the analysts.

### 4.5 Noise and bias errors at individual level

Noise and bias errors are generally considered in team‐oriented decision making. It is also possible to identify noise and bias errors with respect to a single decision maker by considering the decisions she/he made over time. In the present research, noise and bias errors at the individual level were identified by analyzing the scores successively provided by an analyst in different points in time during the same scoring session. As indicated earlier, noise and bias errors at the individual level were measured using the nonparametric statistic Kendall's tau (see Equation (7)). Noise errors were then defined as the level of agreement between the scores assigned at two successive points in time. The results (see Table 3) indicate that group feedback further reduces the noise errors.

Bias errors were defined as the level of agreement between the scores assigned at a given time point and the actual threat level. As underlined earlier, the actual threat levels have been defined by the authors based on information provided by the senior intelligence expert who designed the scenario. The figures in Table 3 indicate that the use of DRSA reduces the bias errors and indicates that group feedback further reduces the bias errors. The results also show that DRSA is slightly more effective in reducing noise errors than bias errors.

**FIGURE 5:**
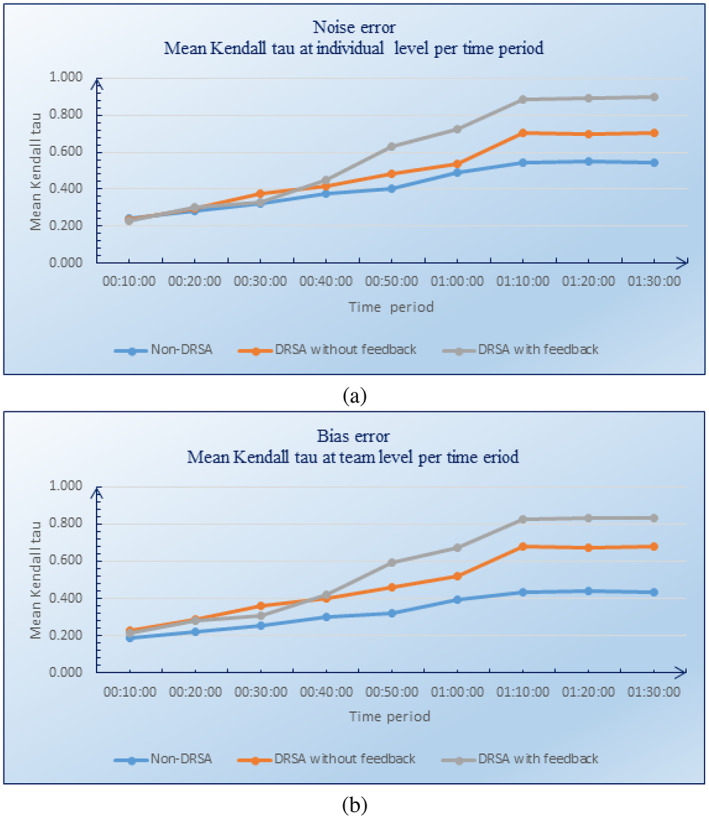
Evolution over time of noise (a) and bias (b) errors at individual level

We also evaluated the evolution over time of noise and bias errors at the individual level. The results are shown in Figure 5a,b, respectively. These figures indicate that the individual agreement level over time in the case of the DRSA with feedback group is higher than the agreement level within the DRSA without feedback group, and much higher than the agreement level within the non‐DRSA group.

Based on these results, we can conclude that the use of DRSA significantly reduces noise and bias errors. This is because a basic assumption in DRSA is that if we have two reports $R_{1}$ and $R_{2}$ such that the evaluations of $R_{1}$ on all the attributes are equal to or worse than those of report $R_{2}$, then report $R_{1}$ should be assigned a higher risk level than report $R_{2}$. Based on this assumption, the DRSA can identify two types of noise error that occur (a) when two reports with same description (i.e., with the same values for all attributes) are assigned to two different risk levels, and (b) when two reports, one of which is worse than the other on all attributes, are assigned the same risk level.

If, during the scoring process, the analysts fail to respect these rules, then the quality of classification will have to be less than unity, indicating the presence of inconsistency. The analysts can then revise their assignments to correct these noise errors. We note that it is easy to correct this type of inconsistency within the developed tool, since the accuracy of the classification is computed after any modification of report scores, so the analyst can identify the cause of the inconsistency on‐the‐fly. If the analyst did not revise her/his assignment, the DRSA can then reduce this error since the basic DRSA (which is implemented in the current tool) identifies the different risk levels of similar reports specified by the analysts and then picks the highest one for all of them.

The identification and measurement of bias errors require the availability of the actual answers (here, scores provided by the expert) but, and as remarked by Kahneman et al. (2016), the actual answers will only be known at the end of the mission, if ever. At this level, we recall that historical decisions, when available, can be used to estimate and correct bias errors, as advocated by Satopää et al. (2021). In the conducted experiment, the actual threat levels had already been determined by the authors based on input from the scenario designer. In practice, however, it is difficult to know the true answer in advance or in real‐time during the scoring process: the true answers will normally be discovered at the end of the mission. Due to this fact, techniques and strategy for the reduction of bias errors should be oriented to the reduction or elimination of the sources that may lead to bias errors instead of trying to handle the bias errors themselves. The developed tool can be enhanced to better anticipate the actual answers in two different ways. First, by enriching the learning set by some relevant historical reports (that should concern missions similar to the one under investigation). These historical reports might serve as benchmarks and could be used by the DRSA to identify the relevant reports faster based on the rules deduced from these historical reports in the beginning of the scoring process. Second, historical reports can be used as testing sets to evaluate the quality of the current scoring process. Both ways can be seen as an implementation of the idea of Satopää et al. (2021) mentioned earlier about using historical decisions to correct bias errors.

### 4.6 Noise and bias errors at team level

**FIGURE 6:**
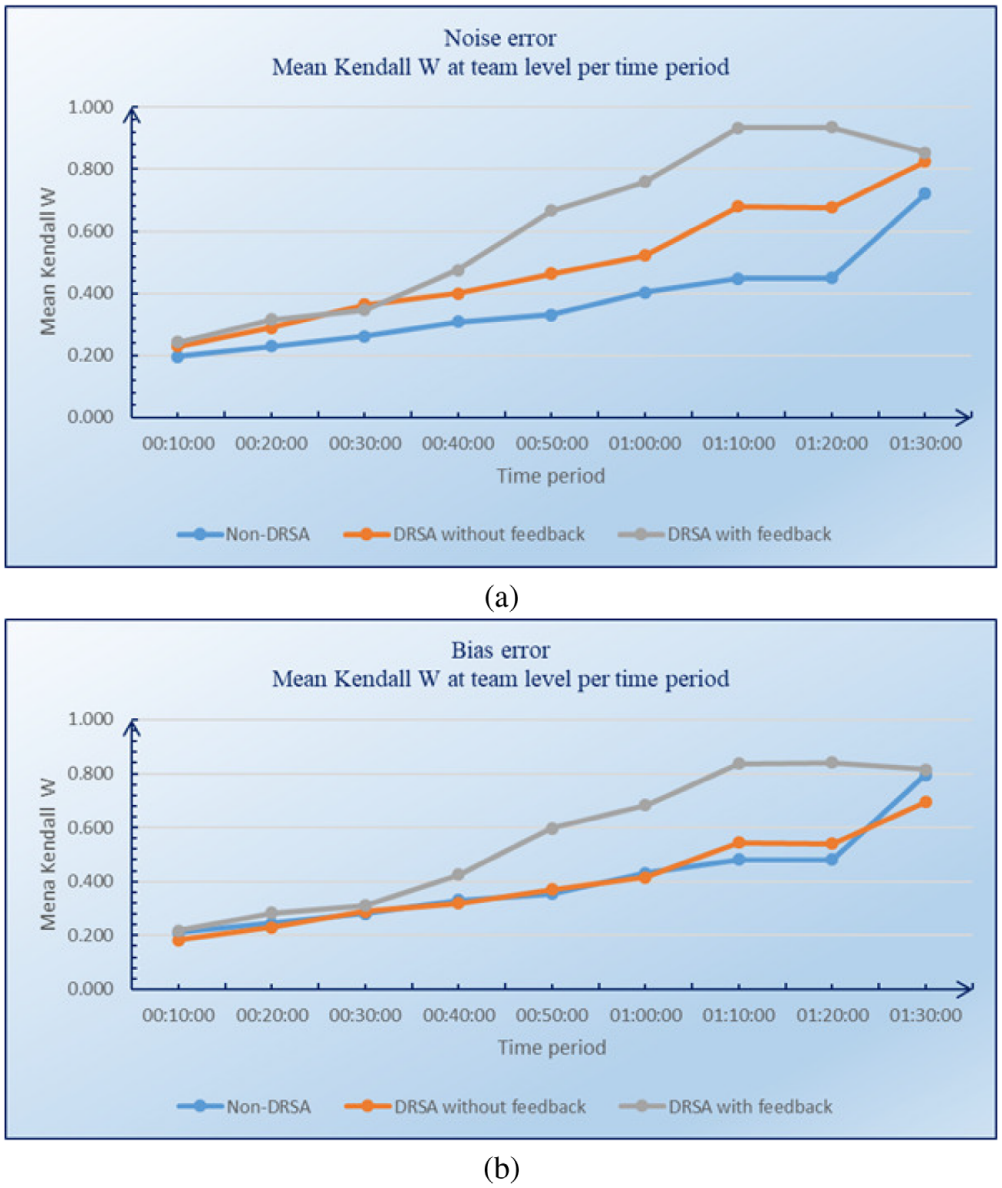
Evolution over time of noise (a) and bias (b) errors at team level

Noise and bias errors at the team level were measured using the nonparametric statistic Kendall's W (see Equation (8)). The results in Table 3 indicate that there was a reduction of noise and bias errors at the team level. In particular, it has been shown that TIDE is slightly more effective at reducing noise errors than bias errors, which confirms the same remark obtained from individual analysts. We also evaluated the evolution over time of noise and bias errors at the team level. The results are shown in Figure 6a,b, respectively. These figures indicate that the team agreement level over time for the DRSA with feedback group is higher than the agreement level within the DRSA without feedback group, and much higher than the agreement level within the non‐DRSA group.

In a previous study by Kerr et al. (1996), it was found that there is no clear or general pattern of relative susceptibility of the judgments of individuals and groups to systematic biases. Relying on a theoretical analysis that uses Davis's (1998) social decision scheme, Kerr et al. (1996) argue that the relative magnitudes of individual and group bias depends upon several factors, such as group size, initial individual judgment, the magnitude of the bias of the individuals, the type of bias, and the group‐judgment process. They also concluded that the social decision scheme offers a framework for specifying some of the conditions under which individuals are both more and less biased than groups. By comparing group and individual performances in a document retrieval task, Wilbur (1998) showed that a group of untrained individuals acting together performed better than any single trained individual and almost at the level of the trained group. In the same direction, Hong et al. (2020) showed that investor opinions extracted from online investment communities confirm the existence of a “crowd wisdom” in financial markets. Recently, Satopää et al. (2021) have shown that teaming reduces bias. According to Satopää et al. (2021), this bias reduction due to teaming may well reflect how the teams were instructed to interact by second‐guessing each other to avoid excessive conformity/herding (Tetlock & Gardner, 2000).

Within TIDE, noise and bias error reduction at the team level is due in part to the use of a majority rule, implemented by the aggregation procedure. Indeed, the aggregation procedure tends to bring together the most frequent decisions and so reduce their dispersion. In the behavioral finance literature, for example, it is emphasized that individual noises and biases tend to be correlated and so cannot be canceled out by aggregation (see, e.g., Shleifer, 2000). This is in contrast with the findings of Yan (2010), that noise and bias can be canceled by aggregation even if they are independent across investors. Following the argument of Yan (2010) but in the military intelligence analysis context, the present paper showed that aggregated scores are less biased than individual scores.

The noise and bias errors reduction at the team level is also due to the “following the crowd” effect, since it has been shown (see, e.g., Hong et al., 2020; Zhang et al., 2014) that individuals in a team‐oriented analysis tend to follow the group (aggregated) decision, which will speed up the processing time. This is in accordance with several studies (e.g., Adomavicius et al., 2009; Bordetsky & Mark, 2000; Robert Jr et al., 2018) that have shown that information feedback can improve the efficiency and effectiveness in individual and team decision making. However, in practice, providing feedback support for real‐time decision making, as in the military intelligence context, may be very difficult. As noted by Lerch and Harter (2001), this is due to the existence of an interdependency between the decisions, which increases the difficulty of the decision making. Furthermore, and as discussed in Liu et al. (2022), the “excess” of group feedback is not a synonym of better decision making at the team level, as the feedback effect will largely depend on its amount, types, and on the level of users' experience of success. In TIDE, group feedback is implemented through the aggregated score. The calculation of the aggregated score relies on the majority rule. Such a simple and well‐known aggregation rule will generally lead to consensual and well accepted decisions, thus accelerating the scoring process. Furthermore, aggregated scores are automatically computed and shared within the team. This will considerably reduce the cognitive process and speed up the overall scoring process.

## 5 THEORETICAL AND PRACTICAL IMPLICATIONS

The main theoretical contribution of the present paper concerns the enhancement of IIS design and use through a series of formal solutions to address noise and bias errors and correct/reduce their effect on the effectiveness and efficiency of intelligence analysts' judgments and decision making. These solutions rely on the use of a preference learning method, namely DRSA, which, as other machine learning methods, is able to reduce the effect of information overflow and time pressure, as intelligence analysts are asked to check only a limited set of intelligence reports. However, the main attractive feature of DRSA is its ability to deal with situations of inconsistency (see, e.g., Deng et al., 2013), which is due to the use of a dominance relation, which is the most elementary and natural preference relation. As shown in the considered application, automatically detecting and dealing with inconsistent decisions permitted reducing noise and bias errors at the individual and team levels. Furthermore, and unlike classical machine learning methods, the DRSA explicitly takes into account the preferences of the analysts and reproduce their behavior through different decision rules (classical machine learning methods assume that the attributes and classes are nonpreference ordered).

Another important feature of TIDE is the support of group decisions, which are objectively combined through the use of majority/veto effect rule, along with the analysts' power. Most of the existing approaches either use a weighted sum‐like method or simple aggregation rules (such as minimum, maximum or average values) to compute aggregated scores. The support of a group decision will improve the intelligence analysis process with respect to two capabilities. First, this will reduce the cognitive effort for senior managers, which will have access to the group decision so as to themselves combine the individual decisions. Second, and as shown by research on the wisdom of crowds (see, e.g., Satopää et al., 2021) and also the current project, groups often make better, more accurate judgments than individuals—even expert individuals. Second, the team decision is used as a group feedback, which improve the efficiency and effectiveness of the individual and team decision making, as shown by several studies (e.g., Adomavicius et al., 2009; Bordetsky & Mark, 2000; Robert Jr et al., 2018). Finally, the timely sharing of aggregated scores with all second line intelligence analysts will speed up the scoring processing and converge more quickly towards a consensus.

This research also contributes to bridging the gap between information behavior and IIS design by showing that preference learning methods along with input from well experienced intelligence analysts about information search, use and decision making play a crucial role in the design of successful and well accepted IIS through a better understanding of information search, use and decision making. For example, the keyword‐based search capability included in TIDE has been extensively discussed with the relevant intelligence experts. Their guidance has greatly shaped the final version of this capability. In particular, the input of intelligence experts was crucial with respect to the type of keywords to be included, how to prioritize the keywords used, and the preferred way to highlight them in Gist fields.

From a practical of point of view, TIDE enhances the manual process by which military intelligence analysts identify high threat emitters. In particular, TIDE supports standard scoring process of SIGINT reports and improves it with respect to several aspects: (a) scoring of intelligence reports relies on a preference learning method, which reduces the processing time by drastically reducing the number of intelligence reports that need to be read and scored; (b) offering a powerful and user‐friendly interface to keyword‐based search capabilities of Gist text, helping analysts filter and prioritize intelligence reports more quickly; (c) capturing and dealing with inconsistent decisions automatically, which will reduce noise and bias errors at the individual and team levels; (d) combining individual scores objectively by using the majority with veto rule; (e) sharing aggregated scores in a timely manner with all second line intelligence analysts, which will speed up the scoring and converge more quickly towards a consensus.

## 6 CONCLUSION

Helping intelligence analysts to be more efficient and more effective in their judgments is crucial for the success of military intelligence missions. Reducing the inconsistency of analysts' judgments, which is mainly due to noise and bias errors, will naturally help analysts to achieve better judgments about the value of the information contained in intelligence reports. In this respect, an innovative decision support tool, the Team Information Decision Engine (TIDE), has been designed, implemented and validated. TIDE is tailored around the Dominance‐based Rough Set Approach (DRSA) into a tool designed to extract and capture the intelligence analysts' interests and behavior. Specifically, the DRSA is able to identify inconsistent judgments and automatically discard them, preventing them from affecting any decision. TIDE is further enhanced by incorporating a majority rule‐based aggregation procedure to combine judgments by individual analysts into consensuses. The team‐based judgments are automatically shared in a timely fashion within the team members, as group feedback, thus reducing the underlying cognitive processes and accelerating the scoring process.

TIDE has been validated through a series of trials in which research participants (acting as analysts) took part in a simulated intelligence assessment task, where the mission was to expose a terrorist attack being planned for a Western European city. A series of measures were used to evaluate the efficiency and effectiveness of the analysts, both at the level of the individual analyst and at the team level. The results showed that the use of TIDE enhanced the effectiveness of the individual intelligence analysis process and considerably improved its efficiency. In particular, the findings show that TIDE made a significant difference by enabling analysts to identify a greater proportion of the relevant reports and filter out irrelevant reports. The results also show that noise and bias errors decrease over time, which is in accordance with previous studies (e.g., Kao et al., 2018) and can be explained by the increasing number of training samples, something which enhances the quality of the induced decision rules and then reduces the number of misclassifications. The results also indicate that the incorporation of team decision‐making support improved both the effectiveness and efficiency of the intelligence analysis process.

Several topics need to be further investigated. The first topic concerns the enhancement of attribute extraction capabilities to better reflect analyst search terms and priorities and result in more accurate rules. Indeed, intelligence reports contain paragraphs of text that need to be read by the analyst. The quality of this text can vary greatly. There will be reports that have minimal relevance to the analysts' mission, while some will contain crucial information. Determining what is important, and how to process the important reports quickly, is the most challenging aspect of the analyst's task. A possible solution to enhance paragraph processing is to use innovative capabilities of text analysis (see, e.g., Chen et al., 2012; Suissa et al., 2022) or through semantic processing instead of simple standalone keywords, by exploiting open‐source software such as UK Defence Science and Technology Laboratory's Baleen (which is freely available from: https://github.com/dstl/baleen3). The latter is an extensible text processing capability that allows entity‐related information to be extracted from unstructured and semi‐structured data sources.

The second topic concerns the support of group feedback. Within TIDE, group feedback is represented by the aggregated score of the intelligence reports. An interesting area for future consideration concerns the employment of more advanced techniques, such as text communication based on synchronicity (Robert Jr et al., 2018) or group facilitator support to enrich the group feedback and, consequently, improve the efficiency and effectiveness of the team decision making. Another aspect related to the computing of the aggregated score concerns the assignment of different levels of “power” to team members. In the current version of TIDE, this “power” level is automatically calculated by the system. TIDE can be enhanced by allowing some authorized users (e.g., mission responsible) to set these powers.

A final important topic concerns the consideration of intelligence data sources. TIDE requires data in a specific format. It can be extended to take any text‐based input (tweets, intercepts, news reports, academic papers, formal reports). Future research could extend TIDE to other nontext types of data. In particular, in recent years there has been a significant drive towards investing in new sensors (e.g., CCTV, UAVs) and collecting open‐source data, which has resulted in significant volumes of data that is challenging to process and extract useful intelligence from in a timely manner. Enhancing TIDE by adding new capabilities for analyzing other types of data than intelligence reports will enlarge its application domain.
